# Supplementary material for: Diagnosis of human brucellosis: Systematic review and meta-analysis
Source: PLoS Negl Trop Dis. 2024 Mar 7;18(3):e0012030. doi: 10.1371/journal.pntd.0012030 (PMC10950246; doi:10.1371/journal.pntd.0012030)
Supplement: S1 File — (DOCX) [file pntd.0012030.s001.docx]

**S1 Table.** Search strategy used in each database.

| **Data base** | **Search strategy** | **File** |
| --- | --- | --- |
| MEDLINE  (by Pubmed) | **Population:**  **#1** (Brucellosis[MeSH Terms]) OR (Brucellosis) OR (Brucelloses) OR (Malta Fever) OR (Fever, Malta) OR (Gibraltar Fever) OR (Fever, Gibraltar) OR (Rock Fever) OR (Fever, Rock) OR (Cyprus Fever) OR (Fever, Cyprus) OR (Brucella Infection) OR (Brucella Infections) OR (Infection, Brucella) OR (Undulant Fever) OR (Fever, Undulant) OR (Brucella abortus) OR (Brucella canis) OR (Brucella melitensis) OR (Brucella ovis) OR (Brucella suis) | 1.492 |
|  | **Index test:**  **#2** (Serologic Tests[MeSH Terms]) OR (Serologic Tests) OR (Serological Tests) OR (Serological Test) OR (Test, Serological) OR (Tests, Serological) OR (Tests, Serologic) OR (Serologic Test) OR (Test, Serologic) OR (Serodiagnosis) OR (Serodiagnoses) OR (Fluorescent Antibody Technique[MeSH Terms]) OR (Fluorescent Antibody Technique) OR (Immunofluorescence) OR (Technics, Fluorescent Antibody) OR (Technic, Fluorescent Antibody) OR (Fluorescent Antibody Technics) OR (Antibody Technics, Fluorescent) OR (Antibody Technic, Fluorescent) OR (Fluorescent Antibody Technic) OR (Techniques, Immunofluorescence) OR (Immunofluorescence Techniques) OR (Immunofluorescence Technique) OR (Immunofluorescence Technics) OR (Immunofluorescence Technic) OR (Antibody Technique, Fluorescent) OR (Antibody Techniques, Fluorescent) OR (Enzyme-Linked Immunosorbent Assay[MeSH Terms]) OR (Enzyme-Linked Immunosorbent Assay) OR (Assay, Enzyme-Linked Immunosorbent) OR (Assays, Enzyme-Linked Immunosorbent) OR (Enzyme Linked Immunosorbent Assay) OR (Enzyme-Linked Immunosorbent Assays) OR (Immunosorbent Assay, Enzyme-Linked) OR (Immunosorbent Assays, Enzyme-Linked) OR (ELISA) OR (Agglutination Tests[MeSH Terms]) OR (Agglutination Tests) OR (Agglutination Test) OR (Test, Agglutination) OR (Tests, Agglutination) OR (Rose Bengal[MeSH Terms]) OR (Rose Bengal) OR (Bengal, Rose) OR (Rapid Diagnostic Tests[MeSH Terms]) OR (Rapid Diagnostic Tests) OR (Diagnostic Test, Rapid) OR (Diagnostic Tests, Rapid) OR (Rapid Diagnostic Test) OR (Rapid Diagnostics) OR (Diagnostic, Rapid) OR (Diagnostics, Rapid) OR (Rapid Diagnostic) OR (Molecular Diagnostic Techniques[MeSH Terms]) OR (Molecular Diagnostic Techniques) OR (Diagnostic Technique, Molecular) OR (Diagnostic Techniques, Molecular) OR (Molecular Diagnostic Technique) OR (Technique, Molecular Diagnostic) OR (Techniques, Molecular Diagnostic) OR (Molecular Diagnostic Technics) OR (Diagnostic Technic, Molecular) OR (Molecular Testing) OR (Testing, Molecular) OR (Polymerase Chain Reaction[MeSH Terms]) OR (Polymerase Chain Reaction) OR (Polymerase Chain Reactions) OR (Reaction, Polymerase Chain) OR (Reactions, Polymerase Chain) OR (PCR) |  |
|  | **Outcome:**  **#3** ((sensitiv*[Title/Abstract] OR sensitivity and specificity[MeSH Terms] OR diagnose[Title/Abstract] OR diagnosed[Title/Abstract] OR diagnoses[Title/Abstract] OR diagnosing[Title/Abstract] OR diagnosis[Title/Abstract] OR diagnostic[Title/Abstract] OR diagnosis[MeSH:noexp] OR (diagnostic equipment[MeSH:noexp] OR diagnostic errors[MeSH:noexp] OR diagnostic imaging[MeSH:noexp] OR diagnostic services[MeSH:noexp]) OR diagnosis, differential[MeSH:noexp] OR diagnosis[Subheading:noexp])) OR ((specificity[Title/Abstract]))    **#4** #1 AND #2 AND #3 AND humans[Filter] |  |
| EMBASE | **Population:**  **#1** ('brucellosis'/exp OR 'brucella infection' OR 'brucella melitensis infection' OR 'malta fever' OR 'mediterranean fever (brucellosis)' OR 'brucellosis' OR 'infection by brucella' OR 'infection by brucella melitensis' OR 'infection due to brucella' OR 'infection due to brucella melitensis' OR 'melitococcosis' OR 'undulant fever' OR 'brucella'/exp OR 'brucella' OR 'brucella contamination' OR 'brucella abortus'/exp OR 'brucella abortus' OR 'bacterium abortus' OR 'brucella abortus bang bacteria' OR 'brucella abortus sensitivity' OR 'brucella canis'/exp OR 'brucella melitensis'/exp OR 'brucella melitensis' OR 'micrococcus melitensis' OR 'brucella ovis'/exp OR 'brucella ovis' OR 'brucella suis'/exp OR 'brucella melitensis biovar suis' OR 'brucella melitensisbv. suis' OR 'brucella suis') | 2.604 |
|  | **Index test:**  **#2** ('serology'/exp OR 'serologic reaction' OR 'serologic specificity' OR 'serologic survey' OR 'serologic technique' OR 'serologic test' OR 'serologic tests' OR 'serological characteristic' OR 'serological specificity' OR 'serological test' OR 'serology' OR 'immunofluorescence'/exp OR 'i.f.' OR 'immune fluorescence' OR 'immune fluorescence technique' OR 'immunofluorescence' OR 'immunofluorescence technique' OR 'immunofluorescent technique' OR 'indirect immunofluorescence' OR 'enzyme linked immunosorbent assay'/exp OR 'elisa' OR 'enzyme labeled immunosorbent assay' OR 'enzyme linked immunoassay' OR 'enzyme linked immunosorbent assay' OR 'enzyme linked immunospecific assay' OR 'enzyme-linked immune assay' OR 'enzyme-linked immuno-assay' OR 'enzyme-linked immunosorbent assay' OR 'agglutination test'/exp OR 'agglutination reaction' OR 'agglutination test' OR 'agglutination tests' OR 'agglutinin test' OR 'test, agglutination' OR 'rose bengal'/exp OR 'bengal rose' OR 'bengal rose stain' OR 'bengal rose staining' OR 'rose bengal' OR 'rose bengal b' OR 'rose bengal disodium' OR 'rose bengal sodium' OR 'rose bengale' OR 'rapid diagnostic test'/exp OR 'molecular diagnosis'/exp OR 'molecular diagnosis' OR 'molecular diagnostic' OR 'molecular diagnostic techniques' OR 'polymerase chain reaction'/exp OR 'pcr (polymerase chain reaction)' OR 'polymerase chain reaction') |  |
|  | **Outcome:**  **#3** ('sensitivity and specificity'/exp OR 'sensitivity and specificity' OR 'specificity and sensitivity' OR 'sensitivity'/exp OR 'specificity'/exp OR 'diagnosis'/exp OR 'bacteriologic diagnosis' OR 'diagnosis' OR 'diagnostic screening' OR 'diagnostic screening programs' OR 'diagnostic sign' OR 'diagnostic tool' OR 'diagnostics' OR 'disease diagnosis' OR 'medical diagnosis' OR 'physical diagnosis' OR 'true positive result'/exp OR 'true negative result'/exp OR 'predictive value'/exp OR 'negative predictive value' OR 'positive predictive value' OR 'predictive value' OR 'predictive value of tests')    **#4** #1 AND #2 AND #3 AND [humans]/lim AND [embase]/lim |  |
| Cochrane Library | **Population:**  **#1** MeSH descriptor: [Brucellosis] explode all trees  **#2** 'brucellosis' OR 'brucella infection' OR 'brucella melitensis infection' OR 'malta fever' OR 'mediterranean fever (brucellosis)' OR 'brucellosis' OR 'infection by brucella' OR 'infection by brucella melitensis' OR 'infection due to brucella' OR 'infection due to brucella melitensis' OR 'melitococcosis' OR 'undulant fever' OR 'brucella' OR 'brucella abortus' OR 'brucella abortus' OR 'bacterium abortus' OR 'brucella abortus bang bacteria' OR 'brucella abortus sensitivity' OR 'brucella melitensis' OR 'brucella canis' OR 'brucella canis' OR 'brucella canidis' OR 'brucella ovis' OR 'brucella ovis' OR 'brucella suis' OR 'brucella melitensis biovar suis' OR 'brucella melitensisbv. suis' OR 'brucella suis' | 27 |
|  | **Index test:**  **#3** MeSH descriptor: [Serologic Tests] explode all trees  **#4** MeSH descriptor: [Fluorescent Antibody Technique] explode all trees  **#5** MeSH descriptor: [Enzyme-Linked Immunosorbent Assay] explode all trees  **#6** MeSH descriptor: [Agglutination Tests] explode all trees  **#7** MeSH descriptor: [Rose Bengal] explode all trees  **#8** MeSH descriptor: [Molecular Diagnostic Techniques] explode all trees  **#9** MeSH descriptor: [Polymerase Chain Reaction] explode all trees  **#10**’serology' OR 'serologic reaction' OR 'serologic specificity' OR 'serologic survey' OR 'serologic technique' OR 'serologic test' OR 'serologic tests' OR 'serological characteristic' OR 'serological specificity' OR 'serological test' OR 'serology' OR 'fluorescent antibody technique' OR 'antibody fluorescent technique' OR 'antibody, fluorescent' OR 'fluorescence antibody technique' OR 'fluorescence antibody test' OR 'fluorescence antiglobulin test' OR 'fluorescent antibody' OR 'fluorescent antibody darkfield method' OR 'fluorescent antibody identification' OR 'fluorescent antibody method' OR 'fluorescent antibody technique' OR 'fluorescent antibody test' OR 'fluorescent inhibition technique' OR 'enzyme linked immunosorbent assay' OR 'rose bengal' OR 'agglutination test' OR 'agglutination reaction' OR 'agglutination test' OR 'agglutination tests' OR 'agglutinin test' OR 'test, agglutination' OR 'molecular diagnostics' OR 'gene expression assay kit' OR 'gene expression test kit' OR 'genetic assay kit' OR 'genetic test kit' OR 'molecular diagnostic test kit' OR 'molecular diagnostics' OR 'polymerase chain reaction' OR 'pcr (polymerase chain reaction)' OR 'polymerase chain reaction'    **#4** (#1 OR #2) AND #3 |  |
|  | **Outcome:**  **#11** MeSH descriptor: [Sensitivity and Specificity] explode all trees  **#12** MeSH descriptor: [Diagnosis] explode all trees  **#13** ('sensitivity and specificity' OR 'sensitivity and specificity' OR 'specificity and sensitivity' OR 'sensitivity' OR 'specificity' OR 'diagnosis' OR 'bacteriologic diagnosis' OR 'diagnosis' OR 'diagnostic screening' OR 'diagnostic screening programs' OR 'diagnostic sign' OR 'diagnostic tool' OR 'diagnostics' OR 'disease diagnosis' OR 'medical diagnosis' OR 'physical diagnosis' OR 'true positive result' OR 'true negative result' OR 'predictive value' OR 'negative predictive value' OR 'positive predictive value' OR 'predictive value' OR 'predictive value of tests')    **#14** (#1 OR #2) AND (#3 OR #4 OR #5 OR #6 OR #7 OR #8 OR #9 OR #10) AND (#11 OR #12 OR #13) |  |
| BVS | **Population:**  **#1** (mh:(Brucelose)) OR (Brucelose) OR (mh:(Brucellosis)) OR (Brucellosis) OR (mh:(Brucelosis)) OR (Brucelosis) OR (Febre Ondulante) OR (Febre de Malta) OR (Infecçãopor Brucella) OR (mh:(Brucella)) OR (Brucella) OR (mh:(Brucella abortus)) OR (Brucella abortus) OR (Bacterium abortus) OR (mh:(Brucella canis )) OR (Brucella canis ) OR (mh:(Brucella melitensis )) OR (Brucella melitensis ) OR (Micrococcus melitensis) OR (mh:(Brucella ovis)) OR (Brucella ovis) OR (mh:(Brucella suis)) OR (Brucella suis)    **Teste index:**  **#2** (Sorologia) OR (Serology) OR (Serología) OR (Serologia) OR (Imunofluorescência) OR (Fluorescent Antibody Technique) OR (Técnica del AnticuerpoFluorescente) OR (Rastreamento de ProteínaFluorescente) OR (Testes de AnticorposFluorescentesAntinucleares) OR (Técnica de Imunofluorescência) OR (Ensaio de ImunoadsorçãoEnzimática) OR (Enzyme-Linked Immunosorbent Assay) OR (Ensayo de InmunoadsorciónEnzimática) OR (ELISA) OR (EnsaioImunoadsorventeEnzima-Associado) OR (EnsaioImunoadsorventeLigado à Enzima) OR (Ensaio de ImunoadsorçãoLigado à Enzima) OR (Aglutinação) OR (Agglutination) OR (Aglutinación) OR (Testes de Aglutinação) OR (Rosa Bengala) OR (Rose Bengal) OR (Técnicas de Diagnóstico Molecular) OR (Molecular Diagnostic Techniques) OR (Técnicas de Diagnóstico Molecular) OR (ReaçãoemCadeia da Polimerase) OR (Polymerase Chain Reaction) OR (Reacción en Cadena de la Polimerasa) OR (PCR) OR (Reação da PolimeraseemCadeia) OR (Reação de PolimeraseemCadeia) OR (ReaçãoemCadeia de Polimerase)  **Outcome:**  **#3** (Sensibilidade e Especificidade) OR (Sensitivity and Specificity) OR (Sensibilidad y Especificidad) OR (Sensibilidade) OR (Especificidade) OR (VerdadeirosNegativos) OR (VerdadeirosPositivos) OR (Valor Preditivo dos Testes) OR (Predictive Value of Tests) OR (Valor Predictivo de las Pruebas) OR (Valor Preditivo) OR (Valor PreditivoNegativo) OR (Valor PreditivoPositivo) OR (Valor Preditivo do Teste) OR (Valores Preditivos de Testes) OR (Reações Falso-Negativas) OR (Reações Falso-Positivas) OR (Diagnóstico) OR (Diagnosis) OR (Detecção) OR (Diagnose)    Bases available after withdrawing MEDLINE in the filter:  **#4** db:("LILACS" OR "IBECS" OR "VETINDEX" OR "BINACIS" OR "LIPECS" OR "CUMED" OR "PAHO" OR "PAHOIRIS" OR "SES-SP" OR "ARGMSAL" OR "BDNPAR" OR "MedCarib" OR "campusvirtualsp_centroamerica")    **#5** #1 AND #2 AND #3 AND #4 | 256 |
| **Total** | | **4.379** |
