# Supplementary material for: Diagnosis of human brucellosis: Systematic review and meta-analysis
Source: PLoS Negl Trop Dis. 2024 Mar 7;18(3):e0012030. doi: 10.1371/journal.pntd.0012030 (PMC10950246; doi:10.1371/journal.pntd.0012030)
Supplement: S2 File — (DOCX) [file pntd.0012030.s002.docx]

**S2 File.** Detail of each index test according to the included studies

1. **Rose Bengal**

| Reference | Index Test | Manufacturer | Antigen species | Case definition | Non-case definition | Case no. | non- case no. | Total no. | TP​ | FN​ | TN​ | FP​ | Sens (%)​ | Spec. (%)​ |
| --- | --- | --- | --- | --- | --- | --- | --- | --- | --- | --- | --- | --- | --- | --- |
| Almashhadany et al., 2022 (29) | Rose Bengal | Torax Biosciences, United Kingdom |  | Culture | Culture | 31 | 40 | 71 | 31 | 0 | 31 | 9 | 100 | 96,9 |
| Araj et al., 1988 (32) | Brucellosis card test | Brewers' Diagnostic Kits, United States | *B. abortus* | Culture | Other diseases | 83 | 72 | 155 | 83 | 0 | 72 | 0 | 100 | 100 |
| Araj et al., 1988 (32) | Brucelloslide test | BioMérieux, France | *B. abortus* | Culture | Other diseases | 83 | 72 | 155 | 83 | 0 | 72 | 0 | 100 | 100 |
| Díaz et al., 2011 (39) | Rose Bengal | Antigen manufactured by Veterinary Laboratory, United Kingdom (>1:4) |  | Culture | Contact with an animal or work accident | 208 | 20 | 228 | 180 | 28 | 20 | 0 | 87,4 | 100 |
| Mert et al., 2003 (50) | Rose Bengal | NR | NR | Culture | NR | 30 | NR | 30 | 30 | 0 | NR | NR | 100 | NR |
| Mizanbayeva et al., 2009 (51) | Rose Bengal | NR | NR | Culture | NR | 63 | NR | 63 | 45 | 18 | NR | NR | 71,4 | NR |
| Nicolleti et al., 1971 (52) | Cart agglutination test | Becton Dickinson, USA | NR | Culture | Culture | 16 | 196 | 212 | 15 | 1 | 154 | 42 | 93,8 | 78,6 |
| Purwar et al., 2016 (57) | Slide agglutination test | Antigen manufactured by Indian Veterinary Research Institute, India | *B. abortus* | Culture | Culture | 20 | 380 | 400 | 20 | 0 | 365 | 15 | 100 | 96,1 |
| Saz et al., 1987 (59) | Rose Bengal | NR | NR | Culture | Culture and conventional tests | 208 | 107 | 315 | 182 | 26 | 107 | 0 | 87,5 | 100 |
| Clavijo et al., 2003 (36) | Brucelloslide or Rose Bengal | BioMérieux, France | NR | Culture and/or SAT | NR | 133 | NR | 133 | 128 | 5 | NR | NR | 96,2 | NR |
| Gomes et al., 2008 (43) | Rose Bengal | Bio Systems, Barcelona, Spain | *B. abortus* | Culture and/or SAT | Healthy | 25 | 90 | 115 | 25 | 0 | 88 | 2 | 100 | 97,7 |
| Marei et al., 2011 (48) | Rose Bengal | Antigen manufactured by Spinreact, Girona, Spain | NR | Culture and/or SAT | Culture and/or SAT | 20 | 30 | 50 | 20 | 0 | 30 | 0 | 100 | 100 |

Legend: *Index test as described in the original article; NR-Not reported; Sens-Sensitivity; Spec-Specificity; TP-True positive; FN-False negative; TN-True negative; FP-False positive.

1. **Standard agglutination test (SAT)**

| Reference | Index Test | Manufacturer | Type of antigen | Title | Case definition | Non-case definition | Case no. | non- case no. | Total no. | TP​ | FN​ | TN​ | FP​ | Sens (%)​ | Spec  (%)​ |
| --- | --- | --- | --- | --- | --- | --- | --- | --- | --- | --- | --- | --- | --- | --- | --- |
| Abdoel et al., 2007 (25) | Seroagglutination test | NR | NR | >1:320 | Culture | NR | 45 | NR | 37 | 8 | NR | NR | 82,22 | NR | NR |
| Akhvlediani et al., 2017 (26) | Wright teste (standard tube agglutination test) | NR | *B. abortus* | >1:200 | Culture | Culture | 33 | 48 | 27 | 6 | 35 | 13 | 81,82 | 72,92 | 72,92 |
| Araj et al., 1988 (32) | Microagglutination test (MAT) | National Veterinary Services Laboratories, Iowa | *B. melitensis* | >1:80 | Culture | Other diseases | 83 | 72 | 83 | 0 | 72 | 0 | 100 | 100 | 100 |
| Díaz et al., 2011 (39) | Seroagglutination test | NR | NR | >1:160 | Culture | Contact with an animal or work accident | 208 | 20 | 160 | 48 | 20 | 0 | 76,9 | 100 | 100 |
| Ertek et al., 2006 (40) | Standard agglutination test | Pendik Veterinary Institute, Istanbul, Turkey | *B. abortus* | >1:160 | Culture | Healthy from endemic area | 32 | 20 | 30 | 2 | 20 | 0 | 93,75 | 100 | 100 |
| Kiel et al., 1987 (46) | Hemagglutination serological test | NR | *B*. *abortus* / *B*. *melitensis* | >1:160 | Culture | NR | 60 | NR | 60 | 0 | NR | NR | 100 | NR | NR |
| Memish et al., 2002 (26) | Standard agglutination test (SAT) | Antigen manufacturer by Wellcome  Diagnostics, England | *B*. *abortus* and *B*. *melitensis* | >1:320 | Culture | Healthy from endemic area | 68 | 70 | 65 | 3 | 70 | 0 | 95,59 | 100 | 100 |
| Mert et al., 2003 (50) | Wright teste (standard tube agglutination test) | Pendik Veterinary Institute, Istanbul, Turkey | *B*. *abortus* | >1:160 | Culture | NR | 30 | 280 | 30 | 0 | 280 | 0 | 100 | 100 | 100 |
| Mizanbayeva et al., 2009 (51) | Wright's serum agglutination test | NR |  | >1:25 | Culture | NR | 63 | NR | 63 | 0 | NR | NR | 100 | NR | NR |
| Mizanbayeva et al., 2009 (51) | Wright's serum agglutination test | NR |  | >1:50 | Culture | NR | 63 | NR | 57 | 6 | NR | NR | 90,48 | NR | NR |
| Mizanbayeva et al., 2009 (51) | Wright's serum agglutination test | NR |  | >1:100 | Culture | NR | 63 | NR | 46 | 17 | NR | NR | 73,02 | NR | NR |
| Mizanbayeva et al., 2009 (51) | Wright's serum agglutination test | NR |  | >1:200 | Culture | NR | 63 | NR | 32 | 31 | NR | NR | 50,79 | NR | NR |
| Nicoletti et al., 1971 (52) | Tube agglutination test | Razi Institute, Hessark, Iran |  | >1:80 | Culture | Culture | 16 | 196 | 16 | 0 | 158 | 38 | 100 | 80,61 | 80,61 |
| Purwar et al., 2016 (57) | Standard tube agglutination test | Antigen manufacturer by Indian Veterinary Research Institute, India | *B*. *abortus* | >80IU/mL | Culture | Culture | 20 | 380 | 20 | 0 | 366 | 14 | 100 | 96,32 | 96,32 |
| Saz et al., 1987 (59) | Standard agglutination test |  | *B*. *abortus* | >1:80 | Culture | Culture and other convention tests | 208 | 107 | 129 | 79 | 107 | 0 | 62,02 | 100 | 100 |
| Xu et al., 2020 (61) | Standard tube agglutination test | Antigen manufacturer by Center for Disease Control and Prevention, China |  | >1:100 | Culture | Other illnesses or healthy | 51 | 338 | 41 | 10 | 316 | 22 | 80,39 | 93,49 | 93,49 |

Legend: *Index test as described in the original article; NR-Not reported; Sens-Sensitivity; Spec-Specificity; TP-True positive; FN-False negative; TN-True negative; FP-False positive.

1. **ELISA**

| Reference | Index Test | Manufacturer | Type of antigen | Case definition | Non-case definition | Case no. | non- case no. | Total no. | TP​ | FN​ | TN​ | FP​ | Sens (%)​ | Spec. (%)​ |
| --- | --- | --- | --- | --- | --- | --- | --- | --- | --- | --- | --- | --- | --- | --- |
| Akhvlediani et al. 2017 (26) | IgG/IgM ELISA | In-house test | NR | Culture | Culture | 33 | 48 | 81 | 32 | 1 | 15 | 33 | 96,97 | 31,25 |
| Akhvlediani et al. 2017 (26) | IgG ELISA | IBL International, Hamburg, Germany | NR | Culture | Culture | 33 | 48 | 81 | 31 | 2 | 27 | 21 | 93,94 | 56,25 |
| Akhvlediani et al. 2017 (26) | IgM ELISA | IBL International, Hamburg, Germany | NR | Culture | Culture | 33 | 48 | 81 | 11 | 22 | 41 | 7 | 33,33 | 85,42 |
| Araj et al., 1988 (32) | IgG ELISA | In-house | *B*. *melitensis* | Culture | Another disease | 83 | 72 | 155 | 83 | 0 | 72 | 0 | 100 | 100 |
| Araj et al., 1988 (32) | IgM ELISA | In-house | *B*. *melitensis* | Culture | Another disease | 83 | 72 | 155 | 83 | 0 | 72 | 0 | 100 | 100 |
| Araj et al., 1988 (32) | ELISA IgA | In-house | *B*. *melitensis* | Culture | Another disease | 83 | 72 | 155 | 83 | 0 | 72 | 0 | 100 | 100 |
| Araj et al., 1990 (33) | IgG ELISA | (National Veterinary Services Laboratories, Ames, Iowa USA). | NR | Culture | Healthy | 21 | 15 | 36 | 21 | 0 | 15 | 0 | 100 | 100 |
| Araj et al., 1990 (33) | IgM ELISA | (National Veterinary Services Laboratories, Ames, Iowa USA). | NR | Culture | Healthy | 21 | 15 | 36 | 21 | 0 | 14 | 1 | 100 | 93 |
| Araj et al., 1990 (33) | IgA ELISA | (National Veterinary Services Laboratories, Ames, Iowa USA). | NR | Culture | Healthy | 21 | 15 | 36 | 20 | 1 | 15 | 0 | 95 | 100 |
| Ayala et al., 2014 (35) | IgA/IgG IELISA® | Harbin Peace River Biotechnology Company Limited, China | NR | Culture | Healthy and negative serology | 49 | 77 | 126 | 48 | 1 | 77 | 0 | 98,0 | 100 |
| Ayala et al., 2014 (35) | IgA/IgG ELISA® rapid | Harbin Peace River Biotechnology Company Limited, China | NR | Culture | Healthy and negative serology | 49 | 77 | 126 | 47 | 2 | 77 | 0 | 95,9 | 100 |
| Ertek et al. 2006 (40) | IgG/IgM ELISA | Novum, Germany | NR | Culture | Healthy from endemic area | 32 | 18 | 50 | 24 | 8 | 17 | 1 | 75 | 94,4 |
| Ertek et al. 2006 (40) | IgG ELISA | Novum, Germany | NR | Culture | Healthy from endemic area | 32 | 20 | 52 | 26 | 6 | 19 | 1 | 81,25 | 95 |
| Ertek et al. 2006 (40) | IgM ELISA | Novum, Germany | NR | Culture | Healthy from endemic area | 32 | 20 | 52 | 32 | 0 | 17 | 3 | 100 | 85 |
| Fadeel et al., 2006 (41) | IgG/IgM ELISA | Antígeno comercial da Beckton Dickinson | *B*. *abortus* | Culture | Negative culture and confirmation of another disease | 202 | 103 | 305 | 196 | 6 | 99 | 4 | 97,03 | 96,12 |
| Mantur et al., 2010 (47) | IgG/IgM ELISA | NovaTec Immundiagnostica GmbH, Dietzenbach, Germany | NR | Culture | Culture | 31 | 72 | 164 | 31 | 0 | 51 | 21 | 100 | 71,31 |
| Memish et al., 2002 (49) | IgG/IgM ELISA | Genzyme Virotech GmbH, Lowenplatz, Russelsheim | NR | Culture | Healthy from endemic area | 66 | 70 | 136 | 62 | 4 | 68 | 2 | 93,9 | 97,1 |
| Memish et al., 2002 (49) | IgG ELISA | Genzyme Virotech GmbH, Lowenplatz, Russelsheim | NR | Culture | Healthy from endemic area | 68 | 70 | 138 | 31 | 37 | 68 | 2 | 45,6 | 97,1 |
| Memish et al., 2002 (49) | IgM ELISA | Genzyme Virotech GmbH, Lowenplatz, Russelsheim | NR | Culture | Healthy from endemic area | 67 | 70 | 137 | 53 | 14 | 70 | 0 | 79,1 | 100 |
| Osoba et al., 2001 (54) | IgG/IgM ELISA | Genzyme Virotech GmbH, Lowenplatz, Russelsheim | NR | Culture | Healthy | 30 | 44 | 74 | 27 | 3 | 44 | 0 | 90 | 100 |
| Osoba et al., 2001 (54) | IgG ELISA | Genzyme Virotech GmbH, Lowenplatz, Russelsheim | NR | Culture | Healthy | 30 | 44 | 74 | 6 | 24 | 44 | 0 | 20 | 100 |
| Osoba et al., 2001 (54) | IgM ELISA | Genzyme Virotech GmbH, Lowenplatz, Russelsheim | NR | Culture | Healthy | 30 | 44 | 74 | 24 | 6 | 44 | 0 | 80 | 100 |
| Peeridogaheh et al. 2013 (56) | IgG/IgM ELISA | Vircell, Spain | LPS by *B*. *abortus* | Culture | Healthy | 11 | 32 | 43 | 11 | 0 | 32 | 0 | 100,00 | 100 |
| Peeridogaheh et al. 2013 (56) | IgG ELISA | Vircell, Spain | LPS by *B*. *abortus* | Culture | Healthy | 11 | 32 | 43 | 9 | 2 | 32 | 0 | 81,82 | 100 |
| Peeridogaheh et al. 2013 (56) | IgM ELISA | Vircell, Spain | LPS by *B*. *abortus* | Culture | Healthy | 11 | 32 | 43 | 8 | 3 | 32 | 0 | 72,73 | 100 |
| Saz et al., 1987 (59) | IgG ELISA | Ag Virion Institute, Switzerland | B. abortus S99 | Culture | Negative culture and negative conventional tests | 208 | NR | 208 | 186 | 22 | NR | NR | 89,42 | NR |
| Saz et al., 1987(59) | IgM ELISA | Ag Virion Institute, Switzerland | B. abortus S99 | Culture | Negative culture and negative conventional tests | 208 | 0 | 208 | 181 | 27 | NR | NR | 87,02 | NR |
| Saz et al., 1987 (59) | IgA ELISA | Ag Virion Institute, Switzerland | B. abortus S99 | Culture | Negative culture and negative conventional tests | 208 | NR | 208 | 167 | 41 | NR | NR | 80,29 | NR |
| Xu et al., 2020 (61) | IgG/IgM ELISA | IBL International, Hamburg, Germany | NR | Culture | Healthy or other disease | 51 | 338 | 389 | 50 | 1 | 333 | 5 | 98,04 | 98,52 |
| Al-Shamahy et al., 1998 (31) | ELISA | *NR* | *B*. *abortus* | Culture and/or SAT | Healthy, other diseases, exposed individuals | 146 | 1891 | 2037 | 64 | 82 | 1846 | 45 | 43,84 | 97,62 |
| Aranís et al., 2008 (34) | IgG ELISA | Vircell, Spain | LPS by *B*. *abortus* | Culture and/or SAT | Confirmation of another disease, negative Culture and SAT- | 10 | 18 | 28 | 8 | 2 | 18 | 0 | 80 | 100 |
| Aranís et al., 2008 (34) | IgM ELISA | Vircell, Spain | LPS by *B*. *abortus* | Culture and/or SAT | Confirmation of another disease, Culture and SAT- | 10 | 18 | 28 | 5 | 5 | 16 | 2 | 50 | 88,9 |
| Clavijo et al., 2003 (36) | IgM ELISA | Laboratorios Vircell, Granada, Spain | NR | Culture and/or SAT | NR | 133 | NR | 133 | 73 | 60 | NR | NR | 54,89 | NR |
| Fadeel et al., 2011 (Egito) (42) | IgG/IgM ELISA | Bio-Quant Brucella | NR | Culture and/or SAT | Healthy or confirmed another disease | 67 | 145 | 212 | 67 | 0 | 50 | 95 | 100 | 34,5 |
| Fadeel et al., 2011 (Egito) (42) | IgG/IgM ELISA | Immuno-Biological Laboratories (IBL) | NR | Culture and/or SAT | Healthy or confirmed another disease | 67 | 145 | 212 | 67 | 0 | 143 | 2 | 100 | 98,6 |
| Fadeel et al., 2011 (Egito) (42) | IgG/IgM ELISA | Vircell | NR | Culture and/or SAT | Healthy or confirmed another disease | 67 | 145 | 212 | 64 | 3 | 143 | 2 | 95,5 | 98,6 |
| Fadeel et al., 2011 (Egito) (42) | IgG/IgM ELISA | Euroimmun | NR | Culture and/or SAT | Healthy or confirmed another disease | 67 | 145 | 212 | 67 | 0 | 130 | 15 | 100 | 89,7 |
| Fadeel et al., 2011 (Estados Unidos) (42) | IgG/IgM ELISA | Bio-Quant Brucella | NR | Culture and/or SAT | Healthy or confirmed another disease | 119 | 38 | 157 | 119 | 0 | 4 | 34 | 100 | 10,5 |
| Fadeel et al., 2011 (Estados Unidos) (42) | IgG/IgM ELISA/IgM | Immuno-Biological Laboratories (IBL) | NR | Culture and/or SAT | Healthy or confirmed another disease | 119 | 38 | 157 | 118 | 1 | 37 | 1 | 99,2 | 97,4 |
| Fadeel et al., 2011 (Estados Unidos) (42) | IgG/IgM ELISA | Vircell | NR | Culture and/or SAT | Healthy or confirmed another disease | 119 | 38 | 157 | 86 | 33 | 37 | 1 | 72,3 | 97,4 |
| Fadeel et al., 2011 (Estados Unidos) (42) | IgG/IgM ELISA | Euroimmun | NR | Culture and/or SAT | Healthy or confirmed another disease | 119 | 38 | 157 | 118 | 1 | 31 | 7 | 99,2 | 81,6 |
| Gómez e tal., 2008 (43) | IgG ELISA | Serion/ Virion, Wurzburg, Germany | NR | Culture and/or SAT | Healthy | 25 | 90 | 115 | 21 | 4 | 90 | 0 | 84 | 100 |
| Gómez e tal., 2008 (43) | IgA ELISA | Serion/ Virion, Wurzburg, Germany | NR | Culture and/or SAT | Healthy | 25 | 90 | 115 | 24 | 1 | 88 | 2 | 96 | 98 |
| Gómez e tal., 2008 (43) | IgM ELISA | Serion/ Virion, Wurzburg, Germany | NR | Culture and/or SAT | Healthy | 25 | 90 | 115 | 15 | 10 | 90 | 0 | 60 | 100 |
| Hasibi et al., 2013 (45) | IgG/IgM ELISA | Immuno Biological Laboratories Company, Germany | NR | Culture and/or SAT | Healthy or other disease | 56 | 126 | 182 | 42 | 14 | 126 | 0 | 75 | 100 |
| Hasibi et al., 2008 (45) | IgG ELISA | Immuno Biological Laboratories Company, Germany | NR | Culture and/or SAT | Healthy | 37 | 78 | 115 | 33 | 4 | 78 | 0 | 89,2 | 100 |

Legend: *Index test as described in the original article; NR-Not reported; Sens-Sensitivity; Spec-Specificity; TP-True positive; FN-False negative; TN-True negative; FP-False positive.

1. **Rapid diagnostic test**

| Reference | Index Test | Antibody detected | Case definition | Non-case definition | Case no. | non- case no. | Total no. | TP​ | FN​ | TN​ | FP​ | Sens (%)​ | Spec. (%)​ |
| --- | --- | --- | --- | --- | --- | --- | --- | --- | --- | --- | --- | --- | --- |
| Abdoel et al. 2007 (25) | Omega Teknika | IgM | Culture | NR | 45 | NR | 45 | 25 | 20 | NR | NR | 55,6 | NR |
| Abdoel et al. 2007 (25) | Omega Teknika | IgG | Culture | NR | 45 | NR | 45 | 35 | 10 | NR | NR | 77,8 | NR |
| Abdoel et al. 2007 (25) | Omega Teknika | IgM/IgG | Culture | NR | 45 | NR | 45 | 41 | 4 | NR | NR | 91,1 | NR |
| Mizanbayeva et al. 2009 (51) | Organon Teknika Ltd, Ireland | IgM/IgG | Culture | NR | 63 | NR | 63 | 63 | 0 | NR | NR | 100,0 | NR |
| Mizanbayeva et al. 2009 (51) | Organon Teknika Ltd, Dublin, Ireland | IgM | Culture | NR | 63 | NR | 63 | 55 | 8 | NR | NR | 87,3 | NR |
| Mizanbayeva et al. 2009 (51) | Organon Teknika Ltd, Dublin, Ireland | IgG | Culture | NR | 63 | NR | 63 | 50 | 13 | NR | NR | 79,4 | NR |
| Clavijo et al., 2003 (36) | In house LPS-impregnated nitrocellulose strip | IgM | Culture and/or SAT | NR | 133 | NR | 133 | 94 | 39 | NR | NR | 70,68 | NR |
| Marei et al., 2011 (48) | Royal Tropical Institute, Amsterdam, Netherlands | IgG | Culture and/or SAT | Culture and/or SAT | 20 | 30 | 50 | 13 | 7 | 29 | 1 | 65 | 96,7 |
| Marei et al., 2011 (48) | Royal Tropical Institute, Amsterdam, Netherlands | IgM | Culture and/or SAT | Culture and/or SAT | 20 | 30 | 50 | 14 | 6 | 29 | 1 | 70 | 96,7 |
| Marei et al., 2011 (48) | Royal Tropical Institute, Amsterdam, Netherlands | IgM/IgG | Culture and/or SAT | Culture and/or SAT | 20 | 30 | 50 | 19 | 1 | 29 | 1 | 95 | 96,7 |

Legend: NR-Not reported; Sens-Sensitivity; Spec-Specificity; TP-True positive; FN-False negative; TN-True negative; FP-False positive.

1. **Coombs test**

| Reference | Index Test | Manufacturer | Title | Case definition | Non-case definition | Case no. | non- case no. | Total no. | TP​ | FN​ | TN​ | FP​ | Sens (%)​ | Spec. (%)​ |
| --- | --- | --- | --- | --- | --- | --- | --- | --- | --- | --- | --- | --- | --- | --- |
| Abdoel et al. 2007 (25) | Coombs | NR | >1:2560 | Cultura | Other diseases | 45 | NR | 45 | 45 | NR | NR | NR | 100 | NR |
| Peeridogaheh et al. 2013 (56) | Brucellacapt^®^ | (Vircell SL, Granada, Spain) | NR | Cultura | Healthy | 11 | 32 | 43 | 11 | 0 | 31 | 1 | 100 | 96,9 |
| Saz et al. 1987 (59) | Coombs | NR | >1:160 | Cultura | Negative culture and conventional tests | 208 | 107 | 315 | 160 | 48 | 107 | 0 | 76,9 | 100 |
| Aranís et al., 2008 (34) | Brucellacapt^®^ | (Vircell SL, Granada, Spain) | NR | Culture and/or SAT | Other disease and negative conventional tests | 10 | 18 | 28 | 7 | 3 | 18 | 0 | 70 | 100 |
| Gómez e tal., 2008 (43) | Brucellacapt^®^ | (Vircell SL, Granada, Spain) | NR | Culture and/or SAT | Healthy | 25 | 90 | 115 | 25 | 0 | 90 | 0 | 100 | 100 |

Legend: NR-Not reported; Sens-Sensitivity; Spec-Specificity; TP-True positive; FN-False negative; TN-True negative; FP-False positive.

**f) Polymerase Chain Reaction (PCR)**

| Reference | Index Test | Primer (target) / Sample type | Detection limit | Case definition | Non-case definition | Case no. | non- case no. | Total no. | TP​ | FN​ | TN​ | FP​ | Sens (%)​ | Spec. (%)​ |
| --- | --- | --- | --- | --- | --- | --- | --- | --- | --- | --- | --- | --- | --- | --- |
| Al-Ajlan et al. 2011 (27) | Conventional PCR | B4 e B5 (BCSP31)/ Blood | 100 UFC/ml | Culture | Healthy | 89 | 40 | 129 | 64 | 25 | 38 | 2 | 71,9 | 95 |
| Al-Ajlan et al. 2011 (27) | Conventional PCR | B4 e B5 (BCSP31)/ Blood culture | NR | Culture | Healthy | 89 | 40 | 129 | 89 | 0 | 40 | 0 | 100 | 100 |
| Al-Ajlan et al. 2011 (27) | Conventional PCR | B4 e B5 (BCSP31)/ Serum | NR | Culture | Healthy | 89 | 40 | 129 | 48 | 41 | 40 | 0 | 53,9 | 100 |
| Al-Ajlan et al. 2011 (27) | Real-time PCR | B4 e B5 (BCSP31)/ Blood | 50  UFC/ ml | Culture | Healthy | 89 | 40 | 129 | 69 | 0 | 40 | 0 | 77,5 | 100 |
| Al-Ajlan et al. 2011 (27) | Real-time PCR | B4 e B5 (BCSP31)/ Blood culture | NR | Culture | Healthy | 89 | 40 | 129 | 89 | 20 | 40 | 0 | 100 | 100 |
| Al-Ajlan et al. 2011 (27) | Real-time PCR | B4 e B5 (BCSP31)/ Serum | NR | Culture | Healthy | 89 | 40 | 129 | 54 | 35 | 40 | 0 | 60,7 | 100 |
| Al-Nakkas et al. 2005 (30) | Conventional PCR (Nested) | (IS711)/ Blood | NR | Culture | Other diseases | 89 | 244 | 333 | 89 | 0 | 244 | 0 | 100 | 100 |
| Nimri et al. 2003 (53) | Conventional PCR | (16S RNA)/ Blood | NR | Culture | Culture and/or SAT | 20 | 25 | 45 | 20 | 0 | 25 | 0 | 100 | 100 |
| Queipo-Ortuno et al. 1997 (58) | Conventional PCR | B4 e B5 (BCSP31)/ Blood | 10 fg | Culture | Other disease or exposed or healthy | 35 | 60 | 95 | 35 | 0 | 59 | 1 | 100 | 98,3 |
| Dal et al., 2018 (37) | Real-time PCR | (BCSP31)/ Serum | 80 UFC/mL | Culture | Culture | 36 | 117 | 153 | 35 | 1 | 61 | 56 | 97,22 | 52,14 |
| Debeaumont et al., 2005 (38) | Real-time PCR | BCSP31 fw e rv (BCSP31)/ Serum | 18 fg | Culture | Healthy or with other illnesses | 17 | 60 | 77 | 11 | 6 | 60 | 0 | 64,7 | 100 |
| Patra et al., 2019 (55) | Real-time PCR | (virB4)/ Blood and others | 1000 fg | Culture | Other infectious diseases | 61 | 54 | 107 | 42 | 19 | 54 | 0 | 68.9 | 100 |
| Zhao et al., 2020 (62) | Real-time PCR | JPF e JPR (OMP-2)/ Blood | 1000 fg | Culture | Culture | 46 | 62 | 108 | 45 | 1 | 49 | 13 | 97,83 | 79,03 |
| Vrioni et al., 2004 (60) | PCR-ELISA | B4 e B5 (BCSP31)/ Blood or Serum | NR | Culture | NR | 179 | NR | 179 | 177 | 2 | NR | NR | 98,9 | NR |
| Vrioni et al., 2004 (60) | PCR-ELISA | B4 e B5 (BCSP31)/ Blood | NR | Culture | NR | 179 | NR | 179 | 167 | 12 | NR | NR | 93,3 | NR |
| Vrioni et al., 2004 (60) | PCR-ELISA | B4 e B5 (BCSP31)/ Serum | NR | Culture | NR | 179 | NR | 179 | 159 | 20 | NR | NR | 88,8 | NR |
| Al-Attas et al., 2000 (28) | Conventional PCR | B4 e B5 (BCSP31)/ Blood | 30 fg | Culture and/or SAT | Culture and SAT [other diseases (18); exposed (9); healthy (5)] | 14 | 33 | 46 | 14 | 0 | 29 | 4 | 100 | 87,5 |
| Hasibi et al., 2008 (45) | Conventional PCR | B4 e B5 (BCSP31)/ Blood | NR | Culture and/or SAT | Healthy Controls | 37 | 78 | 115 | 15 | 22 | 78 | 0 | 45,5 | 100 |
| Marei et al., 2011 (48) | Conventional PCR | B4 e B5 (BCSP31)/ Serum | NR | Culture and SAT | Culture and SAT | 20 | 30 | 50 | 17 | 3 | 29 | 1 | 85 | 96,7 |
| Nimri et al. 2003 (53) | Conventional PCR | (16S RNA)/ Blood | NR | Culture and/or SAT | Culture and SAT | 140 | 25 | 165 | 120 | 20 | 25 | 0 | 85,7 | 100 |

Legend: NR-Not reported; Sens-Sensitivity; Spec-Specificity; TP-True positive; FN-False negative; TN-True negative; FP-False positive.
