# Supplementary material for: Diagnosis of human brucellosis: Systematic review and meta-analysis
Source: PLoS Negl Trop Dis. 2024 Mar 7;18(3):e0012030. doi: 10.1371/journal.pntd.0012030 (PMC10950246; doi:10.1371/journal.pntd.0012030)

**S3 File.** The sensitivity and specificity of all evaluated index tests for diagnosing human brucellosis, using culture as reference standards.

1. The sensitivity and specificity of the Rosa Bengal for diagnosing human brucellosis, using culture as reference standards..
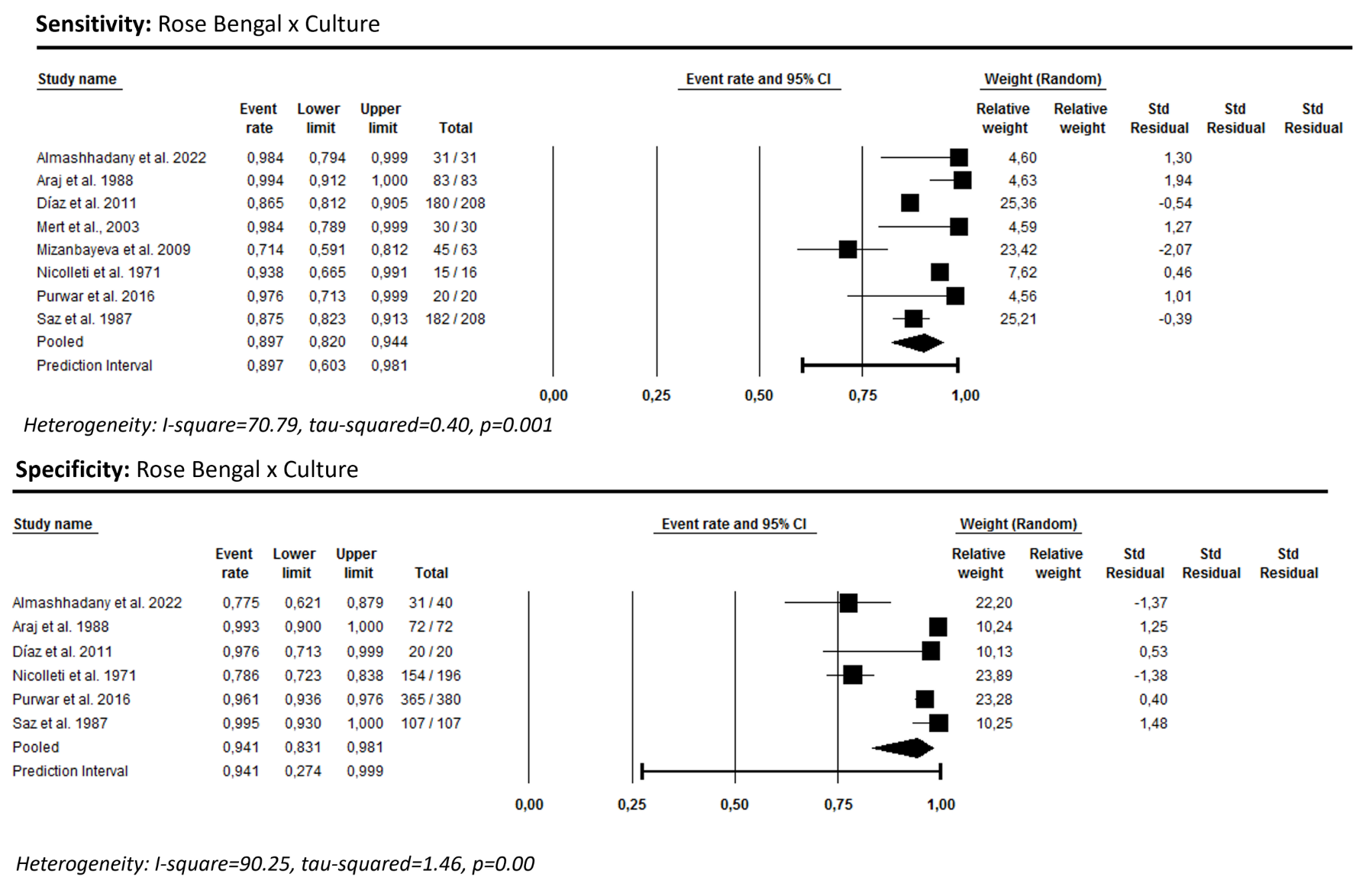


b) The sensitivity and specificity of the SAT for diagnosing human brucellosis, using culture as reference standards.


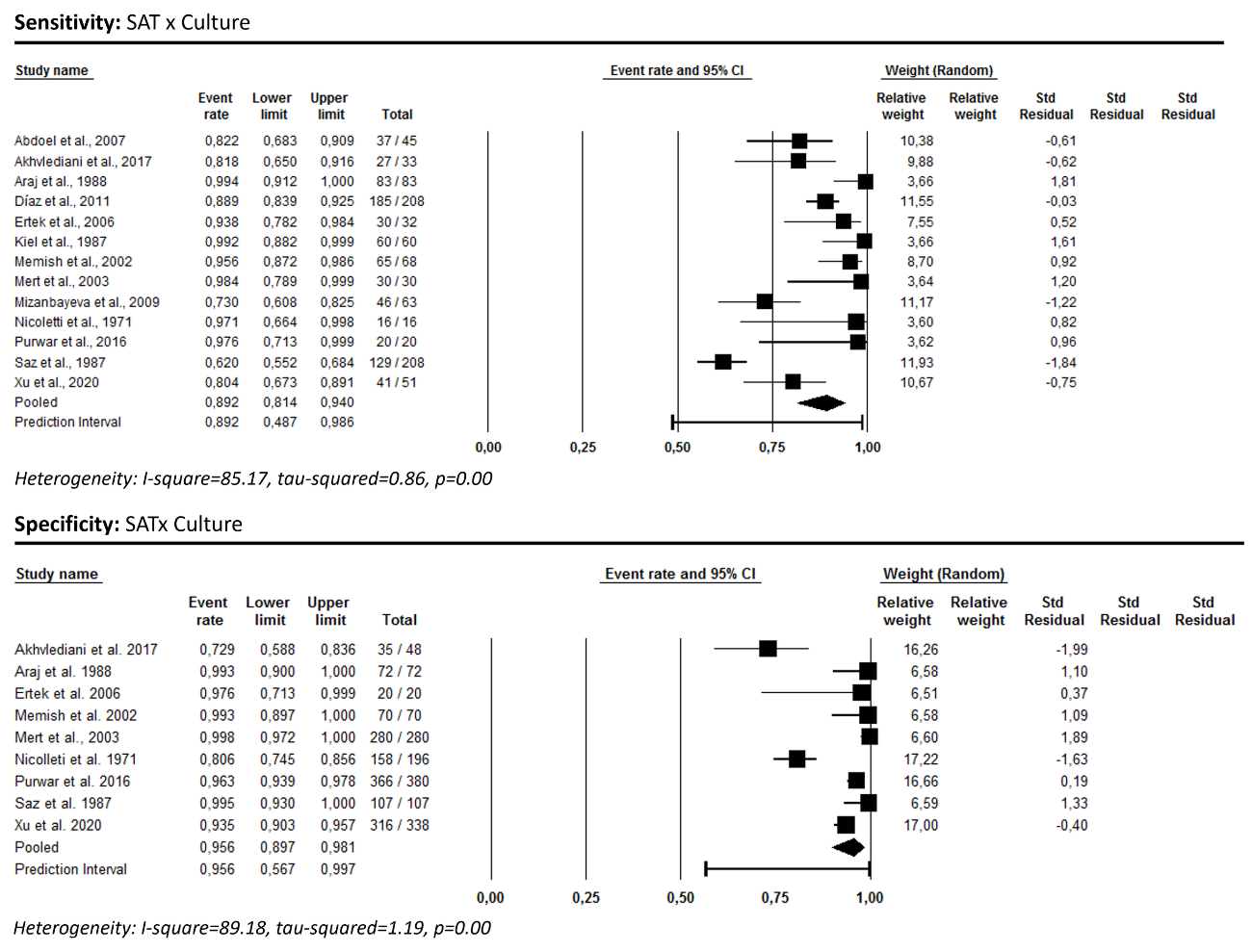


1. The sensitivity and specificity of the ELISA for diagnosing human brucellosis, using culture as reference standards.


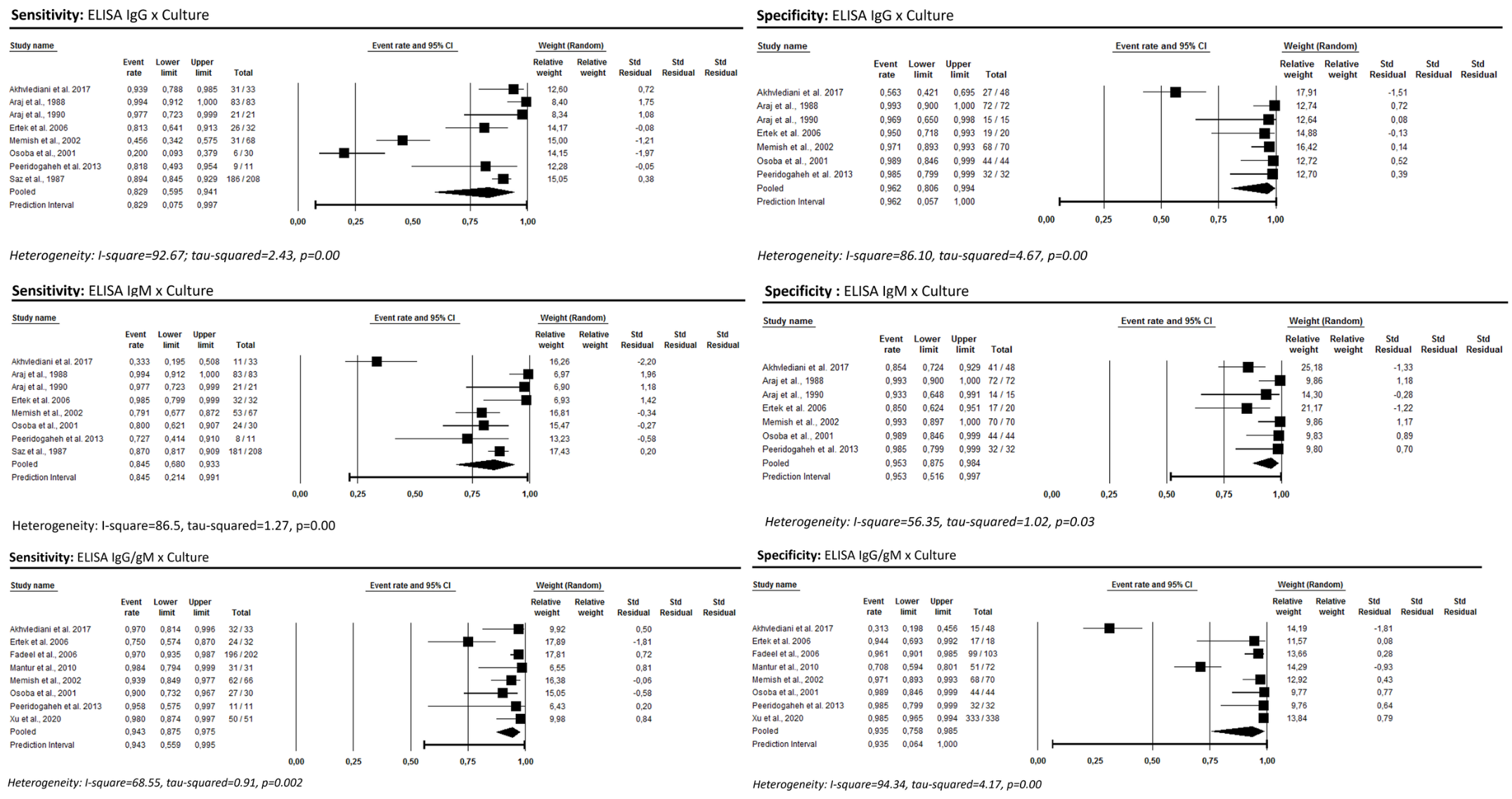


1. The sensitivity and specificity of the Rapid test for diagnosing human brucellosis, using culture as reference standards.


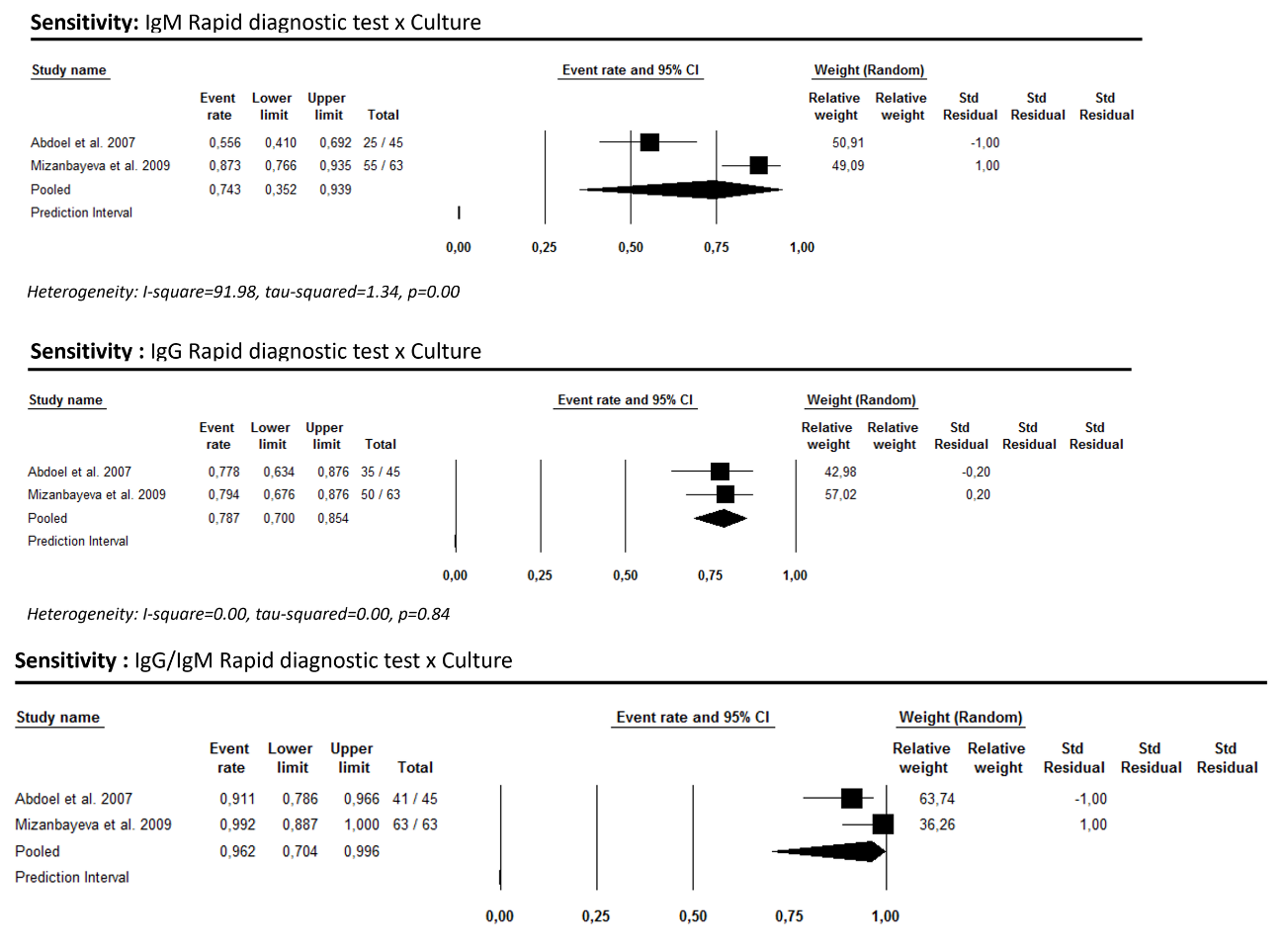


1. The sensitivity and specificity of the Coombs for diagnosing human brucellosis, using culture as reference standards.


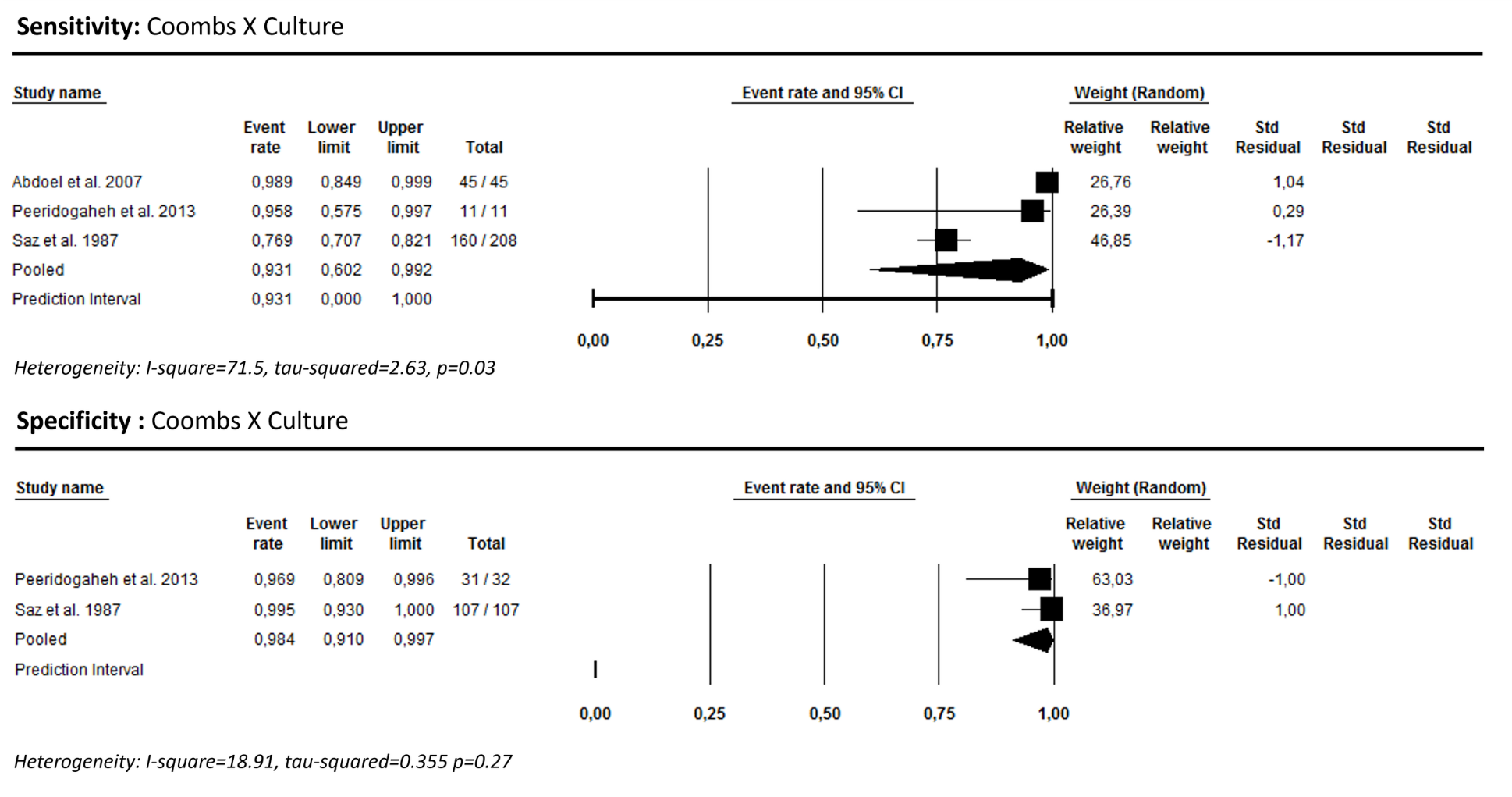


1. The sensitivity and specificity of the Conventional PCR for diagnosing human brucellosis, using culture as reference standards.


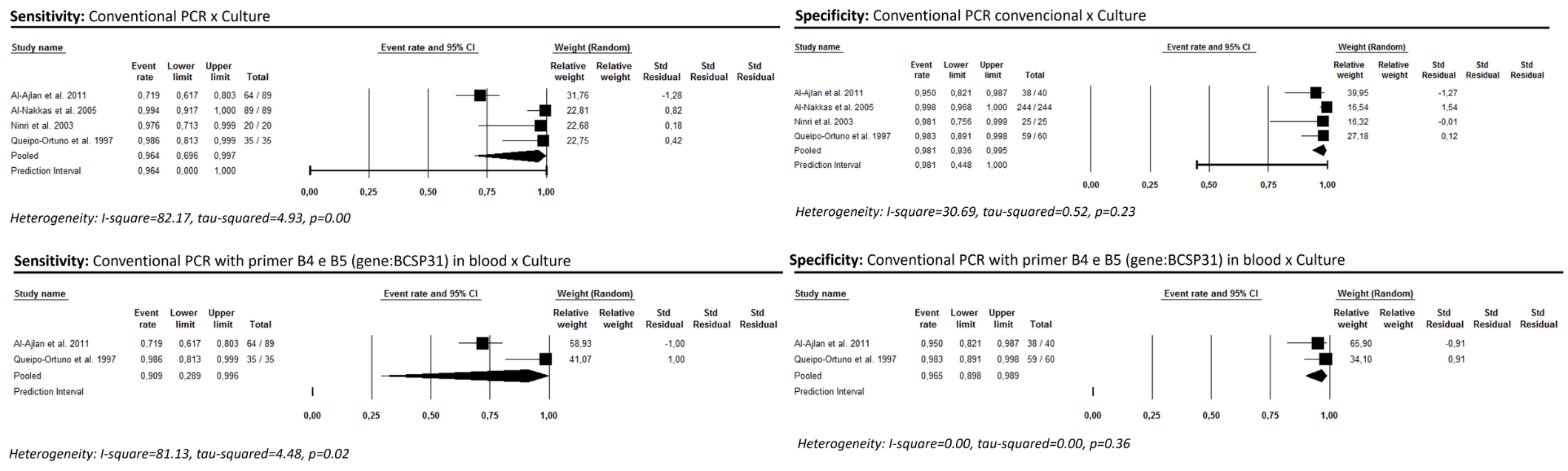


1. The sensitivity and specificity of Real time for diagnosing human brucellosis, using culture as reference standards.


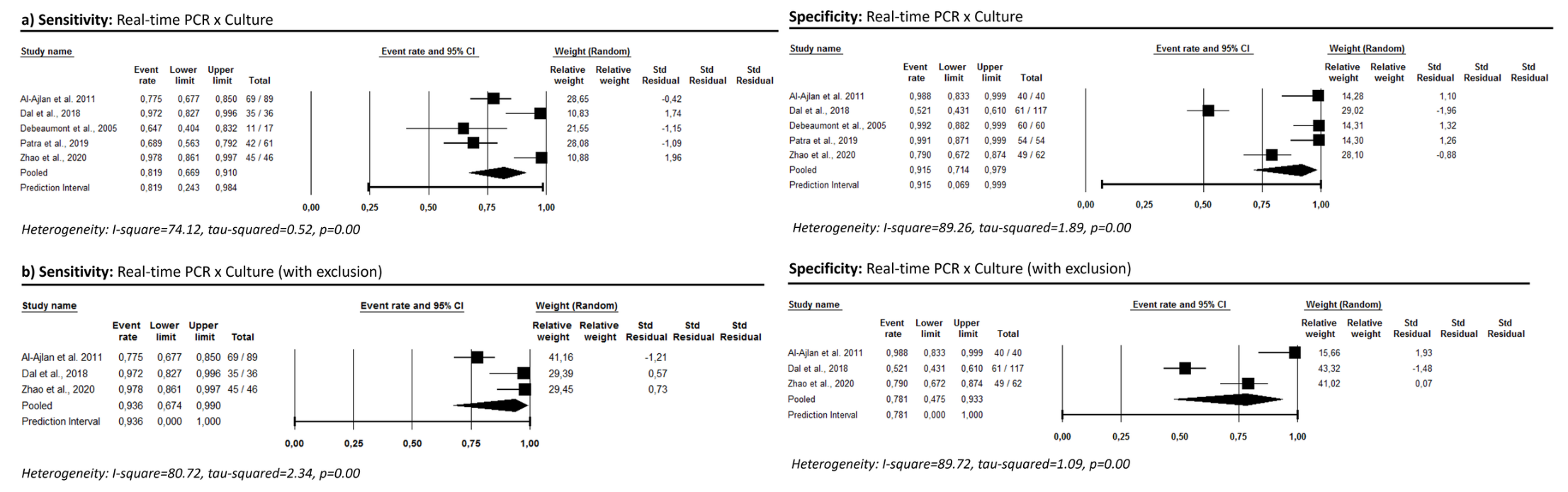

Supplement: S3 File — (DOCX) [file pntd.0012030.s003.docx]
