# Supplementary figures and images for: Diagnosis of human brucellosis: Systematic review and meta-analysis
Source: PLoS Negl Trop Dis. 2024 Mar 7;18(3):e0012030. doi: 10.1371/journal.pntd.0012030 (PMC10950246; doi:10.1371/journal.pntd.0012030)

**S4 File.** Evaluation of the certainty of evidence using the GRADE system

1. Rose Bengal


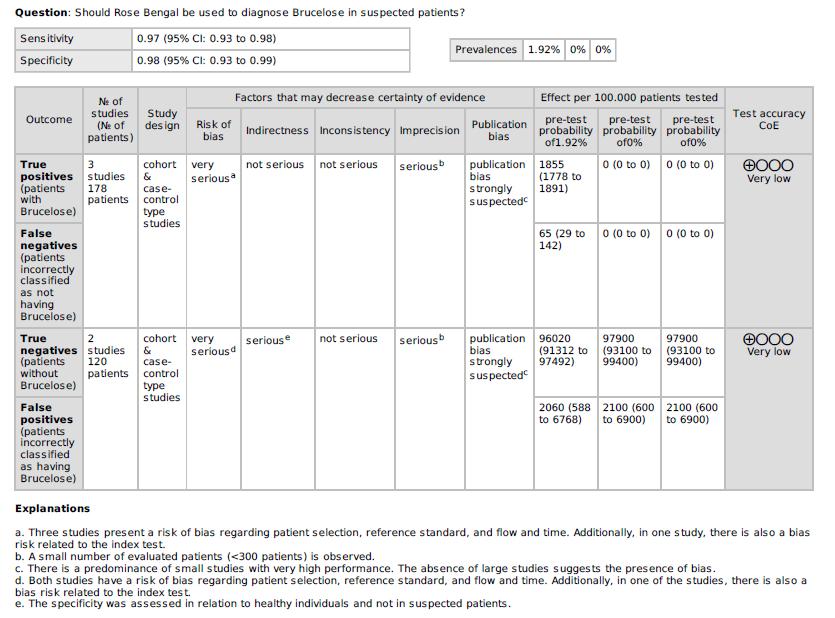


1. Elisa


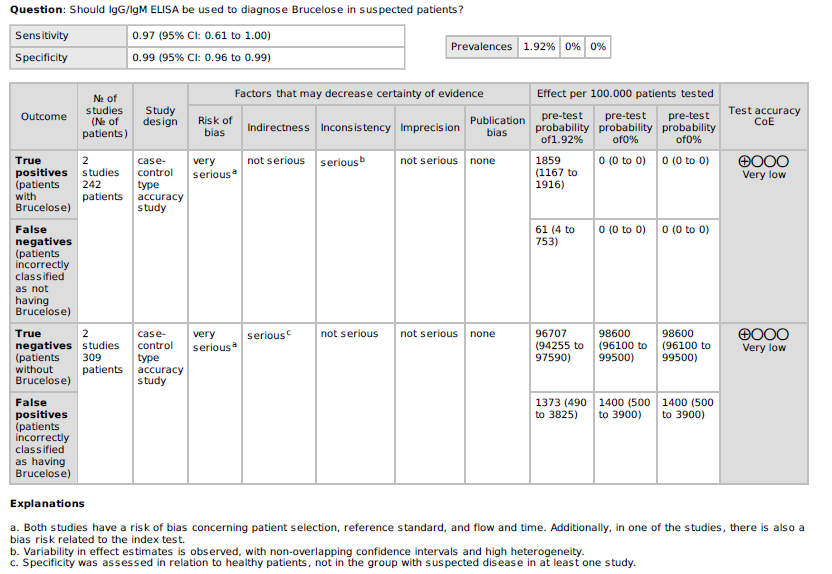


1. PCR


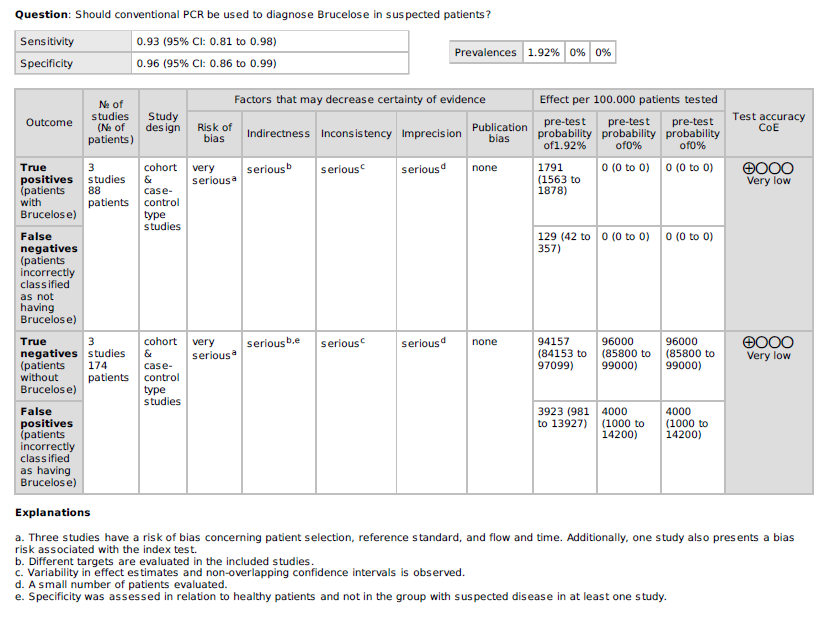

Supplement: S4 File — (DOCX) [file pntd.0012030.s004.docx]
