# Supplementary material for: Diagnosis of human brucellosis: Systematic review and meta-analysis
Source: PLoS Negl Trop Dis. 2024 Mar 7;18(3):e0012030. doi: 10.1371/journal.pntd.0012030 (PMC10950246; doi:10.1371/journal.pntd.0012030)
Supplement: Attachment — Submitted filename: Authors_Response_R1.docx [file pntd.0012030.s005.docx]

Dear Editor,

We are grateful to the reviewers for their time and valuable contributions to improve the manuscript **"** **DIAGNOSIS OF HUMAN BRUCELLOSIS: SYSTEMATIC REVIEW AND META-ANALYSIS" (PNTD-D-23-01480).** We have carefully considered all reviewers' concerns and did our best to improve the text accordingly. The modifications implemented in this revised version are summarized below together with specific answers to the questions placed by the reviewers.

Please let us know in case any other improvement in the manuscript becomes necessary.

Sincerely,

Mariana Lourenço Freire

marianalfreire@hotmail.com

**Reviewer #1:**

**1. Despite the review on the diagnosis of brucellosis in humans, however, a broad introduction to the challenges of diagnosis in general and the most recently used technology in diagnosis have to be included; for example, check and cite the following articles: Doi:** [**https://doi.org/10.51585/gjvr.2023.3.0056**](https://doi.org/10.51585/gjvr.2023.3.0056)**, doi: 10.3389/fmicb.2023.1259479. eCollection 2023.**

**Authors’ comment:** Thank you for the comment and reference. We have included more details on the current state of brucellosis diagnosis and its challenges in the introduction (Lines 87-92; 97-100). However, as the focus of this work is human brucellosis, we chose to prioritize review studies and other references that were dedicated to the condition in humans.

**2. Change gram-negative into Gram, line 64.**

**Authors’ comment:** This sentence was rewritten (line 65)

**3. Information in table 1 is arranged based on the authors' names alphabetically; I suggest arranging based on the date of study from recent to the oldest or based on countries or even the test used to be more practical and informed.**

**Authors’ comment:** Thanks for your suggestion, the Table 1 information was reordered according to the reference test and date of the study’s publication.

**4. I suggest dividing the results into serology and molecular or culture based.**

Authors’ comment: The suggestion was accepted, and the results were stratified in serology and molecular tests.

**5. In the discussion, the authors mentioned that “To our knowledge, this is the first systematic literature review of diagnostic methods for human brucellosis,” which is an overestimation as there are some studies that discussed the same issue before. For example, this chapter, which is available at** [**https://doi.org/10.1007/978-981-13-8844-6_16**](https://doi.org/10.1007/978-981-13-8844-6_16)

Authors’ comment: Thank you for the opportunity to discuss this topic. Indeed, there is a vast literature addressing the diagnosis of brucellosis and some narrative reviews on the topic. However, the strength of this study is precisely that it carried out a systematic, broad, and reproducible search. This approach, despite having failed to identify methodological adequate studies to allow assertive conclusions about the comparative accuracy of the tests, could translate the current state of the evidence, showing the gaps and generating hypotheses to guide future studies. To enhance clarity, we have rephrased this sentence (lines 302-304).

**6. Please refer in discussion to the several immune responses and recent advances in the diagnosis and control of brucellosis, which have been published at** [**https://doi.org/10.51585/gjvr.2022.1.0033**](https://doi.org/10.51585/gjvr.2022.1.0033) **Authors’ comment:** Thank you for your suggestion. Although the pragmatic objective of this study is to gather the best available evidence to support decision-making and secondly, to guide the conduct of accuracy studies, in response to the reviewer's request, we include in the discussion how the new systems biology analyses of antigens recognized by human immune responses in brucellosis have identified large numbers of protein antigens with potential for understanding mechanisms of pathogenesis and immune response may point the way toward diagnostic approaches (lines 399 – 405)

**Reviewer #2:**

1. **Overall, your introductory section provides a detailed description of the background information on brucellosis and the direction and purpose of the study. Although various existing diagnostic methods were described, a clear description of why this study was conducted was lacking. Is it that existing diagnostic methods are not sufficient to meet the actual needs, or are there potentially more efficient diagnostic tools that have not yet been widely adopted? The last sentence clearly states the purpose of the study, but it could be further clarified as to how the expected results will solve existing problems or improve the current diagnostic process.**

Authors’ comment: Thank you for the opportunity to improve the manuscript introduction. We have tried to make clearer the existing gaps and our goals with this systematic review lines (Lines 87-92; 97-100).

2. **Although it is mentioned that keywords were used for the search, no specific list of keywords or search equations are provided in this section. Providing a complete search strategy would help others replicate the findings and enhance the transparency of the study.**

Authors’ comment: As we mentioned in lines 119-120, in Supplementary file 1 the search strategy used in each database included in the present study is available.

3. **Have you conducted sensitivity analyses after excluding each sample separately, and what was the effect of each exclusion on the overall outcome indicator, please provide an overview in the text.**

Authors’ comment: The approach adopted to assess heterogeneity was to compare the heterogeneity among studies evaluating the same type of test considering two different reference tests. Once the reference test was set, our approach was to compare and discuss the convergence or discrepancy between the effects of various studies and to explore the reasons for the trends observed. Sensitivity analysis by excluding studies one by one was not performed because as a random strategy it would not add information to the explanatory analysis adopted.

4. **You need to have a discussion about the inclusion of healthy individuals as a control group, explaining how this practice affects the interpretation of diagnostic test performance.**

Authors’ comment: Thank you for the opportunity to discuss this point. Although the inclusion of healthy individuals in the control group is a common practice, it poses a significant risk of data overestimation. These individuals, not eligible for a brucellosis diagnosis in real-world scenarios, may not accurately reflect the specificity. To verify the occurrence of cross-reactivity it is essential to include symptomatic patients with other alternative confirmed diagnosis. Now, this information was described in the lines 369-373

5. **In the discussion, please further elaborate on the specific problem of incomplete reference standards and their potential impact on the study's conclusions.**

Authors’ comment: The suggestion was accepted, and this information was added in the discussion lines 376-388

6. **If possible, explore the limitations that geographical constraints may impose on the study.**

Authors’ comment: In fact, infectious diseases tests’ performance may differ significantly among geographic regions. This may be due to several factors, such as the prevalence of the disease, differences in the disease presentation or in the infectious agent, difference in the host response and operational aspects related to the test execution, among other factors. Most of the studies gathered were carried out in the Asian countries (24/30), the region with the highest incidence of this disease and with a quite homogeneous and specific population. This information was added to the text (lines 427-434).
