# Supplementary material for: Diagnosis of human brucellosis: Systematic review and meta-analysis
Source: PLoS Negl Trop Dis. 2024 Mar 7;18(3):e0012030. doi: 10.1371/journal.pntd.0012030 (PMC10950246; doi:10.1371/journal.pntd.0012030)
Supplement: Attachment — Submitted filename: Authors_Response_R2.docx [file pntd.0012030.s006.docx]

Dear Editor,

We are grateful to the reviewer for their time and contributions to improve the manuscript " DIAGNOSIS OF HUMAN BRUCELLOSIS: SYSTEMATIC REVIEW AND META-ANALYSIS" (PNTD-D-23-01480). We have carefully considered the reviewer's concern and did our best to improve the text accordingly. The modifications implemented in this revised version are summarized below together with answer to the question placed by the reviewer.

Please let us know in case any other improvement in the manuscript becomes necessary.

Sincerely,

Mariana Lourenço Freire

marianalfreire@hotmail.com

**Reviewer #1:**

**1. The methodology needs to be included in the abstract, despite being well-written in the method section. Please provide a brief explanation of how and when the data was collected, including the criteria for collecting data, the time period, and any exclusion criteria used.**

Thank you for your suggestion, the methodology with the inclusion and exclusion criteria of the systematic review was presented in more detail in the abstract.
